# Evaluation of the scholastic performance of students in 12 programs from a private university in the south-west geopolitical zone in Nigeria

**DOI:** 10.12688/f1000research.16762.2

**Published:** 2019-10-10

**Authors:** Roseline O. Ogundokun, Marion O. Adebiyi, Oluwakemi C. Abikoye, Tinuke O. Oladele, Adewale F. Lukman, Abidemi E. Adeniyi, Adekanmi A. Adegun, Babatunde Gbadamosi, Noah O. Akande

**Affiliations:** 1 Department of Computer Science, Landmark University, Omu-Aran, Kwara State, Nigeria; 2 Department of Computer Science, Covenant University, Ota, Ogun State, Nigeria; 3 Department of Computer science, University of Ilorin, Ilorin, Kwara State, Nigeria

**Keywords:** Academics, Performance, Students, Science and Engineering, Private University, Programmes

## Abstract

Cumulative grade point average (CGPA) is a system for calculation of GPA scores and is one way to determine a student's academic performance in a university setting. In Nigeria, an employer evaluates a student's academic performance using their CGPA score. For this study, data were collected from a student database of a private school in the south-west geopolitical zone in Nigeria. Regression analysis, correlation analysis, and analysis of variance (F-test) were employed to determine the study year that students perform better based on CGPA. According to the results, it was observed that students perform much better in year three (300 Level) and year four (400 Level) compared to other levels. In conclusion, we strongly recommend the private university to introduce program that will improve the academic performance of students from year one (100 level).

## Introduction

Grade Point Average (GPA) and Cumulative Grade Point Average (CGPA) are two concepts usually made mentioned in the education domain and they signified the diverse systems of awarding undergraduates’ scores based upon their educational performances in their different subjects ( Quora, 2019). GPA is computed by summing the total scores and then divide by the credits hours offered by the student. CGPA is the average of the GPA of a particular student which he had acquired in an institution in the courses he had offered. It is also computed by summing the scores acquired by students and then dividing by the computation of his overall credit units. GPA is normally computed in a distinct semester while CGPA is computed for the all-inclusive period of a program in an institution and for students to acquire a good CGPA, the good GPA in all the years must have been obtained by students ( Quora, 2019).

In the white-collar job market now, there is high competition among young graduates. Academic performance is one indicator that highlights university students’ qualification and this is mostly measured using the cumulative grade point average (CGPA). Most employers use CGPA to screen out candidates searching for jobs, and candidates with a higher CGPA are selected ( Yogendra & Andrew, 2017). Therefore, the performance of students ( Oladele *et al*., 2019) in universities should be a concern not only to administrators and educators but also to corporations in the labor market.

Students have to place greater effort in their study to obtain a good grade in order to fulfil the demands of an employer and this makes academic achievement the main factor considered by employers in the recruitment of workers, especially newly graduated students ( Yogendra & Andrew, 2017). The objective of the present study is to determine the study year that students perform better academically across 12 programs in a private university in the south-west geopolitical zone in Nigeria. This study observed that there are few references on the impact of GPA on students’ overall performance and that was the gap filled in this study.

### Literature review

Cullen *et al.* (1996) shows that students’ tertiary academic performance is not influenced by their gender. Erdem *et al.* (2007) tried to establish which social-economics and demographic factors have effects on students’ cumulative grade point average. The authors surveyed at Gaziosmanpaşa University and the targeted population were grade four students. They found out that factors such as the high school a student graduated from, sex, parent’s academic level, and the reading period influence the grade point average of undergraduates. The study of Soto & Anand (2012) shows that student attendance and GPA had significant effects on student performance. Mlambo (2012) opined that gender, age, learning preferences and entry qualification did not cause any significant variation in the academic performance of a student. Rajandran *et al.* (2015) considered the influence of age, sex, race, post-secondary level result and place of origin on the academic performance. 2013/2014 year one students of the University of Malaysia, Faculty of Economics and Administration was the case study. One hundred students were sampled using cross-tabulation and multinomial logistic regression. Results revealed that the effect of gender and place of origin are insignificant while students’ entry qualification have significant effects on the CGPA of the year one students. Foen *et al.* (2016) examined the influence of time used among students to educational accomplishment. The authors observed that the time exhausted on unrelated school undertakings has a negative relationship with CGPA. Fadelelmoula (2018) revealed that lecture attendance affects undergraduates’ performance.

## Methods

Primary data was extracted from Covenant University’s student database ( John *et al*., 2018). The dataset contains the cumulative grade point averages (CGPA) from the first to the fourth year of study and the overall CGPA of students.

IBM Statistical Package for Social Sciences (IBM 20) was used to analyze the data of the scholastic performance of students in 12 programs at the College of Science and Engineering within the year 2010 to 2014. The statistical methodology includes regression analysis, analysis of variance (ANOVA), and descriptive statistics ( Lukman *et al*., 2018).

Approval to use the data was obtained from the Ethical Committee of Landmark University, which is affiliated with Covenant University.

## Results

**Figure 1.**
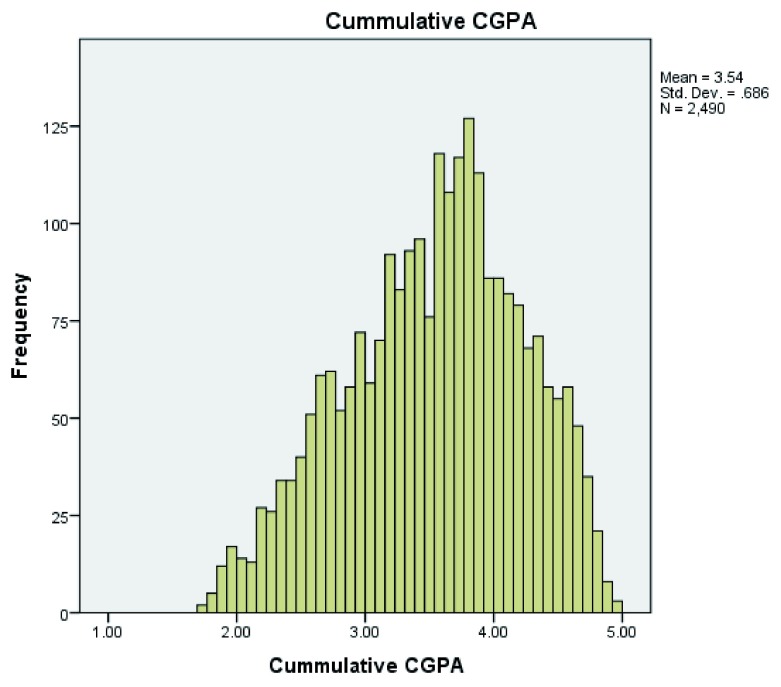
Histogram for students’ cumulative grade point averages between 2010 and 2014 at a private university in Nigeria.

A total of 12 programs were assessed, which included 2490 students. The frequency distribution of the number of students who attended the twelve (12) programs and their graduation years are depicted in Table 1 and Table 2, respectively. The descriptive statistics are provided in Table 3. The results show that the mean performance of all the students at each of the level is not too different from each other. Figure 1 shows a histogram of the cumulative CGPA of students for the years 2010–2014. The distribution of the data is skewed to the right which shows that a high number of the students have a CGPA that is between 2 and 5. The number of students with a CGPA that is less than 2 is low.

**Table 1.** Number of students who attended 12 programs at a private university in Nigeria.

Program	Frequency of students (n)	%	Cumulative Percentile
BCH	142	5.7	5.7
CEN	237	9.5	15.2
CHE	214	8.6	23.8
CHM	111	4.5	28.3
CIS	342	13.7	42.0
CVE	167	6.7	48.7
EEE	418	16.8	65.5
ICE	245	9.8	75.3
MAT	61	2.4	77.8
MCB	168	6.5	84.3
MCE	184	7.4	91.7
PET	206	8.3	100.0
Total	2490	100.0	

**Table 2.** Number of students who graduated from a private university in Nigeria between 2010–2014.

Year	Frequency of students (n)	%	Cumulative Percent
2010	439	17.6	17.6
2011	362	14.5	32.2
2012	576	23.1	55.3
2013	636	25.5	80.8
2014	477	19.2	100.0
Total	2490	100.0	

**Table 3.** Descriptive Statistical Table for Program of Study, Graduation Year, Level CGPA and the Cumulative CGPA for 2010–2014.

	N	Minimum	Maximum	Mean		Std. Deviation
	Statistic	Statistic	Statistic	Statistic	Std. Error	Statistic
CGPA100	2490	1.59	5.00	3.7390	.01299	.64831
CGPA200	2490	1.21	5.00	3.3448	.01545	.77112
CGPA300	2490	.63	5.00	3.4353	.01749	.87290
CGPA400	2490	.00	5.00	3.5713	.01594	.79547
CGPA500	2490	1.73	4.99	3.5379	.01374	.68576

Table 4 shows the correlation matrix of the variables. The variables include CGPA 100 level, CGPA 200 level, CGPA 300 level, CGPA 400 level, CGPA 500 level and the overall CGPA. A strong positive and significant relationships exist between CGPA in the different level and the overall CGPA. The coefficient of determination (R ^2^) in Table 5 shows that the cumulative grade point average in each level explained about 98.1% of the variations in the response variable (the overall CGPA). The F-test shows that the overall regression model is significant (P-value=0.000<0.05). It was also observed that each of the variables has a positive and significant impact on the overall CGPA. The performance of the students in 200 level is more significant (See Table 5). The maximum variance inflation factor shows that none of the variables is correlated (See Table 5). Results show that overall performance of each student depends on their academic performance in each level.

**Table 4.** Correlation Analysis output.

		GPA100	GPA200	GPA300	GPA400	CGPA
CGPA100	Pearson Correlation	1	.718 **	.605 **	.583 **	.795 **
	Sig. (2-tailed)		.000	.000	.000	.000
	N	2490	2490	2490	2490	2490
CGPA200	Pearson Correlation	.718 **	1	.788 **	.718 **	.907 **
	Sig. (2-tailed)	.000		.000	.000	.000
	N	2490	2490	2490	2490	2490
CGPA300	Pearson Correlation	.605 **	.788 **	1	.812 **	.911 **
	Sig. (2-tailed)	.000	.000		.000	.000
	N	2490	2490	2490	2490	2490
CGPA400	Pearson Correlation	.583 **	.718 **	.812 **	1	.878 **
	Sig. (2-tailed)	.000	.000	.000		.000
	N	2490	2490	2490	2490	2490
CGPA	Pearson Correlation	.795 **	.907 **	.911 **	.878 **	1
	Sig. (2-tailed)	.000	.000	.000	.000	
	N	2490	2490	2490	2490	2490

**.Correlation is significant at the 0.01 level (2-tailed).

**Table 5.** Regression Analysis results.

Ordinary Least Squares Estimate Dependent variable=CGPA			
Variable	Coefficient	Std error	t-stat (p-value)
C	0.043	0.012	3.762 (0.000)
CGPA100	0.246	0.004	57.846 (0.000)
CGPA200	0.262	0.005	56.271 (0.000)
CGPA300	0.247	0.004	57.003 (0.000)
CGPA400	0.238	0.004	56.371 (0.000)
Diagnostic tests			Statistics
R ^2^			0.981
F-test			31795.426(0.000)
Maximum Variance Inflation Factor			3.933

*P-value in the parenthesis.

## Conclusion

In this report, we have analyzed the performance of students in 12 programs at a private university in Nigeria. From the various analysis carried out, it was observed that a large number of students graduated in 2013, and from the 12 programs students of electrical and electronic engineering have the highest percentage of graduate students. The descriptive statistics show that the mean performance of all the students at each of the level is not too different from each other. The performance of the student at each level is pivotal to their overall CGPA. In conclusion, we strongly recommend the private university to introduce program that will improve the academic performance of students from year one (100 level).

## Data availability

Zenodo: Dataset on the academic performance of students in 12 programmes from a private university, http://doi.org/10.5281/zenodo.1482513 ( Oluwaseun *et al.*, 2018).
