# Supplementary material for: Evaluation of the scholastic performance of students in 12 programs from a private university in the south-west geopolitical zone in Nigeria
Source: F1000Res. 2019 Oct 10;8:154. Originally published 2019 Feb 5. [Version 2] doi: 10.12688/f1000research.16762.2 (PMC6798315; doi:10.12688/f1000research.16762.2)
Supplement: Peer review history

# Peer review: Reviewer response for version 2

**DOI:** 10.5256/f1000research.22947.r54977

**Published:** 2019-10-21

**Authors:** Semiu Akanmu

**Affiliations:** 1 North Dakota State University, Fargo, USA; 2 Augustine University, Ilara_Epe, Lagos, Nigeria

I acknowledged the fact that the authors have attended to (some) of the suggested revisions. However, there are still three issues with the current revised version of the work. Two are major, while one is minor.

 The minor issue is the use of Quora as a bibliographical reference. I am not sure if the publisher has a lenient provision for this. But I do not think Quora's posts can pass peer review or sound publishing practice tests. I will rather advise alternative verifiable materials, even if it is university handbook for the definitions of GPA and CGPA.

 The major issues are (a) literature review and (b) conclusion sections of the paper.

 The literature review section, as it is currently, is bibliography listing: A said this, B said this, and C reported this. This is listing of bibliography not reviewing of literature. In review, we will want to see what do(es) A, B, and C have in common and what differentiate them -the constructs studied, demography/sample analyzed and results reported. Review must synthesize the literature, highlight similarity or/and differences, and what might account for these, from the authors' perspectives. This is what is called scholarly footing, which must inevitably form the foundation of the new study.

 The conclusion section, unbelievably, did not include the discussion part, and not in any distinct section of the article. The discussion part, either as a distinct section or part of the conclusion section is very important. This is where the findings of your study are situated within those of past related works (which you would have reviewed in the literature review section). It is expected to show what you have done differently as a way of providing different context for previous similar studies, complementing previous studies, or contending previous findings.

I confirm that I have read this submission and believe that I have an appropriate level of expertise to confirm that it is of an acceptable scientific standard, however I have significant reservations, as outlined above.

# Peer review: Reviewer response for version 2

**DOI:** 10.5256/f1000research.22947.r54978

**Published:** 2019-10-17

**Authors:** Robert G. Carroll

**Affiliations:** 1 Brody School of Medicine, East Carolina University, Greenville, NC, USA

Thank you for addressing my concerns.

I confirm that I have read this submission and believe that I have an appropriate level of expertise to confirm that it is of an acceptable scientific standard.

# Peer review: Reviewer response for version 1

**DOI:** 10.5256/f1000research.18322.r44048

**Published:** 2019-08-09

**Authors:** Raheela Asif

**Affiliations:** 1 Department of Computer Science & Software Engineering, N.E.D University of Engineering & Technology, Karachi, Pakistan

**Is the work clearly and accurately presented and does it cite the current literature?**

 Literature review doesn't (directly) lead to the work of this study. Authors should provide an extensive and detailed literature review, which demonstrates their extensive knowledge in the research field and the recognition of the existing achievements in the area.

**Is the study design appropriate and is the work technically sound?**

 The authors should also explain the main differences between their research and the previous achievements.

 The final conclusions provided in the paper are not clearly formulated and correspond to the presented research results. Further steps of the performed research should also be identified by the authors.

I confirm that I have read this submission and believe that I have an appropriate level of expertise to confirm that it is of an acceptable scientific standard, however I have significant reservations, as outlined above.

# Response

**Published:** 2019-10-02

**Authors:** Roseline Ogundokun

**Affiliations:** Landmark University, Nigeria

Literature review doesn't (directly) lead to the work of this study. Authors should provide an extensive and detailed literature review, which demonstrates their extensive knowledge in the research field and the recognition of the existing achievements in the area.[s1] 

 [Response] An extensive and detailed literature review that demonstrates extensive knowledge in this field had been attended to as advised by the reviewer.

The authors should also explain the main differences between their research and the previous achievements.

The final conclusions provided in the paper are not clearly formulated and correspond to the presented research results. Further steps of the performed research should also be identified by the authors.[s1] 

 [Response]These have been attended to as suggested by the reviewer

# Peer review: Reviewer response for version 1

**DOI:** 10.5256/f1000research.18322.r50772

**Published:** 2019-07-05

**Authors:** Semiu Akanmu

**Affiliations:** 1 North Dakota State University, Fargo, USA; 2 Augustine University, Ilara_Epe, Lagos, Nigeria

**Citing relevant literature:**

 I find the in-text citations extremely scanty. The introduction which should weave the justification(s) of the study in specific terms and how it intends to attend to either a practical problem or/and a theoretical problem is inadequate. Explanations provided in the Introduction section generally addressed why "the performance of students should be of concern", not specifically why an investigation of the relationship between the study year and student grade should be conducted. Perhaps, and this should be justified in the literature, it is (a) to be able to design a level-specific intervention program that would support academic performance, (b) to serve as foundation for future studies that would identify factors that contribute to the positive or negative influence of a study level on the student grade.

 There is a slight mention of this in the concluding sentence, but more emphasis on the "why this study?" should be made known at the Introduction section.

 In the same vein, there should be clearer definitions of GPA and CGPA (on level basis and as overall), their relationship, probably in mathematical terms.

**Design of the study, methodology and data analysis:**

 The concluding part of the Introduction section reads that " The objective of the present study is to determine the study year that students perform better academically across 12 programs" which best informs the choice of descriptive and the correlation analysis. Regression is therefore unnecessary because it accounts for influence of a variable on the other, and it must be with adequate attention to all latent and confounding variables.

 I also did not see ANOVA, though mentioned in the methods, in the data analysis. If it would be needed, there should thorough reading of its justification and how best it suits this work. I will suggest reading Julie Pallant's Guide to Using SPSS for further insights.

 Also, Variance Inflation Factor (VIF) is wrongly interpreted. Therefore, this statement "The maximum variance inflation factor shows that none of the variables is correlated" cannot be correct because (a) it contradicts an earlier statement that "A strong positive and significant relationships exist between CGPA in the different level and the overall CGPA", and (b) does not explain variance, which is square of the standard deviation, in view of understanding the variables' multi-collinearity.

 Note that correlation is synonymous to relationship, and it does not mean Variance in statistical terms.

**Discussion and Conclusion:**

 The conclusion, since it ordinarily relied heavily on the data analysis and literature cannot be said to be adequate. There are no past related studies to situate the findings of this study and establish the fresh insights it attempts bring forth. This requires a Discussion section. A better analysis and inferences would then address the concluding section.

**Required corrections:**

1. Review 5 -10 past related studies to partly support your introduction, especially the justification of the study in specific terms, and as literature to situate your findings in a discussion section.
2. Stick to "finding relationship", thus, descriptive  correlation analyses would be sufficient. These, however, must be done thoroughly with report on data treatment and cleansing, and justification of the suitability of the statistical method.

I confirm that I have read this submission and believe that I have an appropriate level of expertise to confirm that it is of an acceptable scientific standard, however I have significant reservations, as outlined above.

# Response

**Published:** 2019-10-02

**Authors:** Roseline Ogundokun

**Affiliations:** Landmark University, Nigeria

I find the in-text citations extremely scanty. The introduction which should weave the justification(s) of the study in specific terms and how it intends to attend to either a practical problem or/and a theoretical problem is inadequate. Explanations provided in the Introduction section generally addressed why "the performance of students should be of concern", not specifically why an investigation of the relationship between the study year and student grade should be conducted. Perhaps, and this should be justified in the literature, it is (a) to be able to design a level-specific intervention program that would support academic performance, (b) to serve as foundation for future studies that would identify factors that contribute to the positive or negative influence of a study level on the student grade.[s1] 

 [Response]All these suggestions have been attended to by the authors

There is a slight mention of this in the concluding sentence, but more emphasis on the "why this study?" should be made known at the Introduction section.

In the same vein, there should be clearer definitions of GPA and CGPA (on level basis and as overall), their relationship, probably in mathematical terms.

[Response] These have been included in the study (manuscript)

The concluding part of the Introduction section reads that " The objective of the present study is to determine the study year that students perform better academically across 12 programs" which best informs the choice of descriptive and the correlation analysis. Regression is therefore unnecessary because it accounts for influence of a variable on the other, and it must be with adequate attention to all latent and confounding variables.[s1] 

 [Response]These have been attended to as suggested by the reviewer

I also did not see ANOVA, though mentioned in the methods, in the data analysis. If it would be needed,[s1] 

 [Response]This reflects in the regression table (F-test). The Anova in regression is used to assess the overall significance of the model.

# Peer review: Reviewer response for version 1

**DOI:** 10.5256/f1000research.18322.r47348

**Published:** 2019-05-01

**Authors:** Robert G. Carroll

**Affiliations:** 1 Brody School of Medicine, East Carolina University, Greenville, NC, USA

This paper describes the grade point average variances in students enrolled in a private university during a 5 year period.  Analysis revealed that grade point averages were not significantly different based on program of study. The cumulative grade point average, a number derived from the grade point average in each year, was significantly correlated with each of the component year grade point averages.

 Tables 1 and 2 include a column for cumulative percent, which does not add any useful information, and can be deleted. The rightward shift in figure 1 may be explained by dismissal of students with low GPAs - but no indication of dismissal/attrition is indicated in the manuscript. The conclusion in the abstract that students perform much better in level 3 and 4 do not appear supported by table 3, and is not mentioned in the Conclusion section of the paper .

I confirm that I have read this submission and believe that I have an appropriate level of expertise to confirm that it is of an acceptable scientific standard, however I have significant reservations, as outlined above.

# Response

**Published:** 2019-10-02

**Authors:** Roseline Ogundokun

**Affiliations:** Landmark University, Nigeria

This paper describes the grade point average variances in students enrolled in a private university during a 5 year period. Analysis revealed that grade point averages were not significantly different based on program of study. The cumulative grade point average, a number derived from the grade point average in each year, was significantly correlated with each of the component year grade point averages.

Tables 1 and 2 include a column for cumulative percent, which does not add any useful information, and can be deleted. The rightward shift in figure 1 may be explained by dismissal of students with low GPAs - but no indication of dismissal/attrition is indicated in the manuscript. The conclusion in the abstract that students perform much better in level 3 and 4 do not appear supported by table 3, and is not mentioned in the Conclusion section of the paper .[Response] 

 [Response]All these suggestions have been attended to as suggested by the authors and the necessary revision had been made in the manuscript.
